# Minimally Invasive Surgical Techniques for the Treatment of Dogs with Urinary Incontinence Due to Ureteral Ectopia

**DOI:** 10.3390/life15040548

**Published:** 2025-03-27

**Authors:** Przemysław Prządka, Bartłomiej Liszka, Wojciech Krajewski, Sylwester Gerus, Ludwika Gąsior, Agnieszka Antończyk, Piotr Skrzypczak, Dominika Kubiak-Nowak, Mateusz Hebel, Kamil Suliga, Zdzisław Kiełbowicz, Dariusz Patkowski

**Affiliations:** 1 Department and Clinic of Surgery, Faculty of Veterinary Medicine, Wroclaw University of Environmental and Life Sciences, 50-375 Wroclaw, Poland; bartlomiej.liszka@upwr.edu.pl (B.L.); ludwika.gasior@upwr.edu.pl (L.G.); agnieszka.antonczyk@upwr.edu.pl (A.A.); piotr.skrzypczak@upwr.edu.pl (P.S.); dominika.kubiak-nowak@upwr.edu.pl (D.K.-N.); mateusz.hebel@upwr.edu.pl (M.H.); kamil.suliga@upwr.edu.pl (K.S.); zdzislaw.kielbowicz@upwr.edu.pl (Z.K.); 2 Department of Minimally Invasive and Robotic Urology, Medical University of Wroclaw, 50-556 Wroclaw, Poland; wojciech.krajewski@umw.edu.pl; 3 Department of Pediatric Surgery and Urology, Medical University of Wroclaw, 50-369 Wroclaw, Poland; sylwester.gerus@umw.edu.pl (S.G.); dariusz.patkowski@umw.edu.pl (D.P.)

**Keywords:** laparoscopy, surgery, animals, urology, ectopy

## Abstract

Ectopic ureters are uncommon congenital abnormalities in dogs, leading to urinary incontinence. Ectopic ureters can be intramural or extramural. Intramural is usually the more prevalent form. Female dogs are affected more often than male dogs. This disease requires the surgical correction of the ureters. Three surgical techniques have been described in this regard: neoureterostomy, nephroureterectomy, and ureteroneocystostomy. In this study, we present minimally invasive surgical procedures for treating extra- and intramural ectopic ureters. Sixteen client-owned dogs with clinical signs of urinary incontinence due to ectopic ureters underwent surgery. Laparoscopic ureteroneocystostomies were performed to correct three extramural cases and one atypical intramural case of ectopic ureters. Additionally, cystoscopically guided laser ablation was used to correct 12 cases of intramural ectopic ureters. In all cases, the procedures were achieved without the need for conversion to open surgery. Among the minor complications, slight hematuria and a few cases of cystitis, which responded to conservative treatment, were noted. Major postoperative complications were not observed. Only one out of sixteen dogs failed to regain urinary continence, but it responded to pharmacological treatment. In conclusion, cases of ectopic ureters may benefit from minimally invasive surgical techniques when their use is feasible.

## 1. Introduction

Ectopic ureters (EUs) are congenital abnormalities characterized by one or both ureteral orifices being located outside the trigone of the urinary bladder [1]. EUs have anomalous origins, with the ureteral bud migrating along the mesonephric duct [2]. EUs can be categorized as either intramural or extramural [3]. For intramural EUs, connection with the bladder wall is within the anatomically expected place; however, the ureters fail to terminate in the anatomically correct position within the bladder chamber [3]. Intramural EU orifices open within the urethra or lower genital tract due to tunneling through the submucosa [3]. Intramural EUs have a higher incidence rate than extramural EUs, with the former accounting for 64–95% of occurrences in dogs [2,4,5]. Like intramural cases, extramural EUs open into the urethra or lower genital tract; however, the ureters divert around the bladder wall, failing to achieve attachment [1,2]. Ectopic ureters are often linked with other urinary comorbidities, including hydronephrosis, hydroureters, pelvic bladder issues, renal aplasia, renal hypoplasia, ureteroceles, urethral sphincter mechanism incompetence, and ureterovesical abnormalities [1,2]. In dogs, urinary incontinence can be either continuous or intermittent, and this information is recorded by the owner the moment a dog is born or purchased. It is the leading cause of ectopic ureters, with the majority of patients being less than a year old [2,6,7]. It has been reported that female dogs account for a 20-fold greater prevalence than males [2,8].

There are currently three surgical techniques for correcting ectopic ureters: neoureterostomy, nephroureterectomy, and ureteroneocystostomy. In a ureteroneocystostomy, the ectopic ureter must be transected distally, with the proximal segment being anastomosed—through extravesicular or intravesicular techniques, or a combination approach—to the urinary bladder [7,9]. A neoureterostomy requires an incision of the bladder mucosa, covering the intramural ureter wall, and then, the bladder mucosa and ureteral mucosa must be sutured together. To avoid persistent draining via the ectopic submucosal tract, either ligation or resection can be performed on the ectopic segment distal to the newly created opening [7,10,11]. For patients that have non-functioning kidneys or incurable pyelonephritis but a functioning contralateral kidney, nephrectomy is advised [7].

Due to the wide surgical access required to perform open-surgery techniques, such as neoureterostomy and ureteroneocystostomy, the corresponding patient will undergo major abdominal and bladder surgical trauma [7]. Minimally invasive treatment options are available, such as cystoscopically guided laser ablation; however, this option requires practitioners to have appropriate training, as well as access to specialized equipment [12]. Intramural ectopic ureter transection via cystoscopic guidance, similar to cystoscopically guided laser ablation, has been explored [12]. Other minimally invasive alternatives to veterinary surgery (which have been recently published) include performing a ureteroneocystostomy laparoscopically [13], an approach that has been employed in human ureteral transposition procedures for several years [14,15].

In this study, we assumed that it is possible to treat ureteral ectopy using only minimally invasive techniques. Consequently, we performed cystoscopically guided bladder mucosa ablation and laparoscopic ureteroneocystostomy. Dogs with intramural ureteral ectopia were treated via cystoscopic laser ablation, whereas extramural ectopia was treated laparoscopically.

## 2. Materials and Methods

Animal selection: The study group comprised client-owned dogs presenting symptoms of urinary incontinence that had been admitted to the Department and Clinic of Surgery of the Faculty of Veterinary Medicine of Wroclaw University of Environmental and Life Sciences. Every dog that displayed signs of ectopic ureters qualified for minimally invasive surgery. The severity of each dog’s urinary incontinence was recorded in their medical notes, ranging from urine leakage when at rest or during excited moments to continual urinary dribbling. All laparoscopic and cystoscopic surgeries were conducted by operators experienced in the use of both techniques. Prior to commencement, written permission for the surgical study was granted by the Ethics Committee of the Wroclaw University of Environmental and Life Sciences (Faculty of Veterinary Medicine Animal Welfare Advisory Team No. 4.2025). After being informed about the requirement for anesthesia, the procedures, and their associated risks, all the owners signed consent forms.

In this article, we present a retrospective study of the results of treating 16 dogs with ureteral ectopia out of 42 determined through evaluations as having clinical signs of urinary incontinence. This study included dogs with computed tomography-confirmed ureteral ectopia. Dogs with incontinence caused by issues other than ureteral ectopia were not included in this study.

### 2.1. Complementary Tests and Computed Tomography

**Figure 1:**
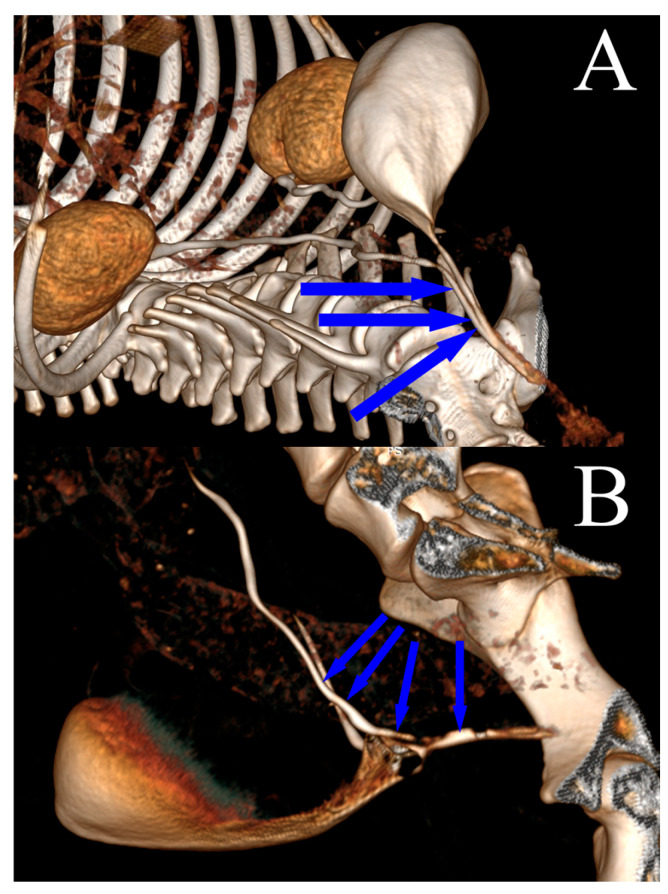
Computed tomography visualization (3D reconstruction) of the ectopic ureter course in a female dog after the administration of intravenous contrast. The blue arrows indicate the course of the ectopic ureter: (**A**) extramural ectopia; (**B**) intramural ectopia.

Complete blood tests (hematological and biochemical), urinalysis, and urine cultures were carried out prior to diagnostic imaging. In the diagnostic examination, diagnostic images, including native and contrast-enhanced computed tomography (CT) images of the abdomen and genitourinary system, were collected (Figure 1A,B).

### 2.2. Surgical Procedures

Based on the results of the diagnostic tests, the dogs with urinary incontinence caused by ectopic ureters were subjected to operations using one of two endoscopic techniques currently available in veterinary medicine. For the dogs presenting with intramural ectopic ureters, cystoscopically guided laser ablation was performed. Laparoscopic ureteroneocystostomies were performed on dogs with extramural ectopic ureters. We decided which endoscopic pathway to follow based on an assessment of computed tomographic excretory urography, performed for each dog with suspected urinary incontinence due to ectopia of the ureter. General anesthesia was administered to every patient. All the procedures were performed by the same surgical team.

### 2.3. Cystoscopically Guided Laser Ablation

For the female dogs, cystoscopically guided laser ablation was performed as described by Berent et al. [6], with modifications, whereas cystoscopically guided laser ablation was performed for male dogs as described by Berent et al. in another study [16].

**Figure 2:**
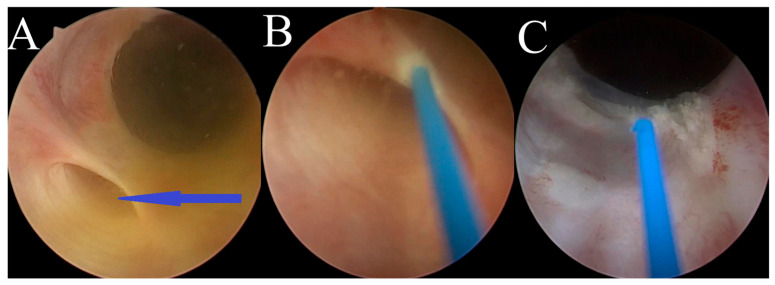
Cystoscopic images of an intramural ectopic ureter in a female dog: (**A**) the ectopic ureter opening into the urethra, indicated by the arrow; (**B**) image captured during cystoscopically guided laser ablation of the ectopic ureter; and (**C**) image captured after cystoscopically guided laser ablation of the ectopic ureter wall.

The female dogs were placed in dorsal recumbency. The skin around the vulva was scrubbed and disinfected with a chlorhexidine solution, and sterile surgical drapes were applied. A rigid ureteronephroscope was inserted into the bladder chamber and ureteral orifice in a retrograde manner, utilizing manual irrigation with saline (0.9% NaCl) solution. An examination of the ureterovesicular junctions was performed, the ectopic ureters were identified, and their locations were confirmed (Figure 2A). A laser fiber (272 μm Hol:YAG—Quanta System, Samarate, Italy) was then inserted into the ectopic ureteral opening through the working channel of the ureteronephroscope (Figure 2B). To correctly angle the tip of the laser fiber with respect to the medial aspect of the ectopic ureteral wall, the tip was deflected toward the urethral lumen. Subsequently, in a pulsating manner, the ectopic ureteral wall was carefully transected at the medial aspect using the Hol:YAG laser. This procedure was considered complete once the orifice of the ureter was within the lumen of the urinary bladder (Figure 2C).

### 2.4. Cystoscopically Guided Laser Ablation in Male Dogs

**Figure 3:**
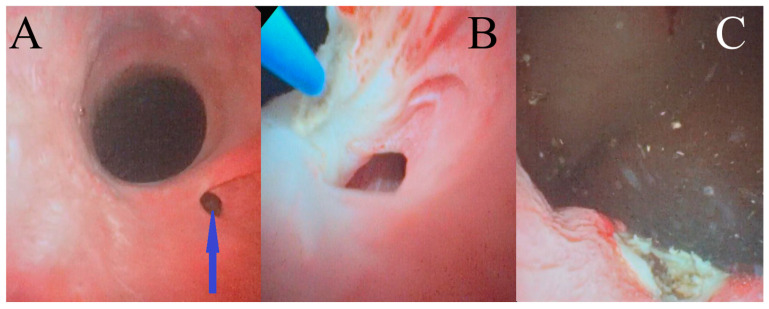
Cystoscopic images of an intramural ectopic ureter in a male dog: (**A**) the ectopic ureter opening into the urethra, indicated by the arrow; (**B**) image captured during cystoscopically guided laser ablation of the ectopic ureter; and (**C**) image captured after cystoscopically guided laser ablation of the ectopic ureter wall.

Male dogs were placed in a right-hand laterally recumbent position. The skin around the penis was scrubbed and disinfected with chlorhexidine solution, and the preputium was rinsed with non-alcohol iodine solution. After sterile surgical drapes were placed, a flexible cystoscope (2.8 mm, Hawk, Zhejiang, China) was passed through the urethral orifice in a retrograde manner, utilizing manual saline irrigation (0.9% NaCl) solution (Figure 3A). The laser fiber (272-μm Hol:YAG) was introduced into the flexible cystoscope’s working channel and guided into the ureteral opening. In order to correctly angle the laser’s fiber tip toward the ureter’s medial wall, the cystoscope was deflected toward the urethral lumen, ensuring that the lateral wall was avoided. Transection of the medial ureter wall was then performed by using the Hol:YAG laser in a pulsating manner (Figure 3B). This procedure was considered complete once the ureterovesicular junction was within the lumen of the bladder (Figure 3C).

### 2.5. Laparoscopic Ureteroneocystostomy

**Figure 4:**
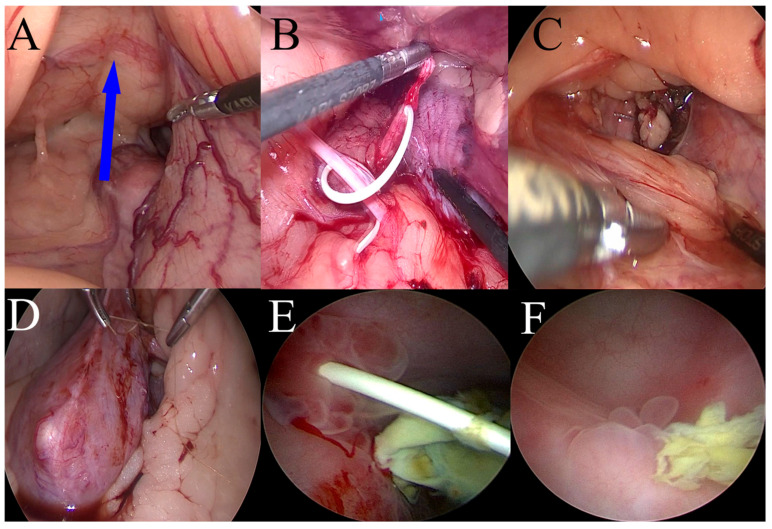
(**A**–**D**) are intraoperative images captured during the laparoscopic ureteroneocystostomy: (**A**) laparoscopic intraoperative view of the ectopic ureter (blue arrow) before dissection from the surrounding tissue. (**B**) ectopic ureter with an intra-ureteral catheter introduced into its lumen. (**C**) ureter, with the intra-ureteral catheter inside, transposed into the bladder lumen; (**D**) intraoperative image captured during the laparoscopic suturing of the ureteral wall to the bladder wall; (**E**,**F**) cystoscopic view of the transposed ureter two weeks after surgery; (**E**) image captured before removal of the ureteral stent; and (**F**) image captured after removal of the ureteral stent.

Laparoscopic ureteroneocystostomies were performed as described by Prządka et al. [13], with modifications. Urethral catheters were placed into each dog’s bladder preoperatively to allow urinary drainage. In order to slightly distend the bladder during each operation, sterile Ringer’s solution was introduced. Catheter removal occurred 24h to 48h postoperatively. The dogs were then placed with their affected (ectopic) side facing up in a laterally recumbent position. Sterile surgical drapes were put in place once the operating field had been antiseptically prepared. Karl Storz SE & Co. KG (Tuttlingen, Germany) and B Braun Aesculap (Tuttlingen, Germany) were the manufacturers of the endoscopic equipment. A 5 mm 30° scope was used for the laparoscopic procedures. Using an open technique, a 5 mm reusable trocar was inserted using a longitudinal incision in the umbilicus to establish pneumoperitoneum (CO_2_). Depending on the size of the dog and the availability of operating space, the insufflation pressure in the abdominal cavity ranged between 8 and 10 mmHg prior to insertion of optics. Next, two 3.5 mm diameter trocars were inserted consecutively in a triangular fashion under the control of the endoscope. The ectopic ureters could be located and identified once access to the abdominal cavity had been achieved (Figure 1A), allowing for careful dissection of the ectopic ureter from the remaining adipose and peritoneal tissues. Sliding-knot suturing techniques were then employed to ligate the ureter at the level of the bladder wall using suture material (polyfilament 2/0, Novosyn, B. Braun, Rubi, Spain). To minimize the risk of bleeding, the blood vessels near the ligature were coagulated. Following that, laparoscopic scissors were used to cut the proximal part of the ureter transversely to its long axis, with the urinary bladder containing the ligated fragment. The free ureteral fragment then underwent spatulation 3–5 mm from the transversely cut edge. Then, a 3Fr intra-ureteral catheter (Double Pigtail Uretal Stent, Cook Medical, Bloomington, IN, USA) was inserted into the ureteral lumen. To achieve this, a ureteral catheter placed on a guide wire was introduced into the abdominal cavity via a 16-gauge injection needle. After the guide wire was placed in the lumen of the ureter, a ureteral catheter was inserted through it (Figure 4B). The next step of the operation required the use of a laparoscopic hook to construct a new orifice proximally to the original opening of the ectopic ureter. By placing the dissected ureter in the planned area for transposition, the new opening could be preselected. The best location for the new orifice could then be chosen, without excessive tension between the tissues at the anastomosis of the ureter wall and bladder. Subsequently, a 4–5 mm segment of ureter, along with the other end of the intra-ureteral catheter, was passed through the newly made opening into the bladder (Figure 4C). The last stage required suturing the ureter wall to the bladder wall, encompassing the serous and muscular layers of the two organs, with 4–6 stitches (polyfilament 5/0, Novosyn, B. Braun, Rubi, Spain) and a sliding-knot technique (Figure 4D). Special care was taken to avoid occlusion of the lumen when suturing through the ureteral wall. Immediate observations of the surgical field were made to check for blood or urinary leakage at the anastomosis site. There was no need for additional trocar placement or conversion to open surgery, as correct placement of the initial trocars, described earlier, enabled the operations to be performed as planned. Single intermittent sutures (monofilament 2/0; Dafilon; B. Braun, Rubi, Spain) were used to close the trocar openings. The duration of all laparoscopic procedures was counted from the first incision to the last skin suture.

### 2.6. Follow-Up Evaluation

To allow for evaluation of the kidneys’ and ureters’ morphologies, as well as the site of the ureterovesical procedure, complete physical examinations and abdominal ultrasounds were recommended at 24 h, 48 h, and six-week intervals after the operations. Additionally, two weeks after the laparoscopic ureteroneocystostomy procedure, the patients underwent cystoscopy to remove the inserted intra-ureteral catheters, and ureterovesical anastomosis was assessed (Figure 4E,F). The owners were regularly contacted for at least six months postoperatively to collect information about the health of the patients.

### 2.7. Statistical Analysis

To enable statistical analysis, Excel software (Microsoft Corp., Version 16.56, Microsoft, Redmond, WA, USA) was utilized. Numbers and percentages are reported for the animals affected by the variables of interest, and the overall results are reported as median and range figures.

## 3. Results

Urinary incontinence due to ectopic ureters was observed in 16 unsterilized dogs (13 females and 3 males) that met the criteria for inclusion in this study. The average weight of the dogs was 18.2 kg (with a range of 8–31 kg), and the average age was 11 months (with a range of 7–24). Through CT observations and urogenital endoscopy results, intramural ectopia of the ureter was identified in 12 dogs (9 females and 3 males), and extramural ureter ectopy was found in 4 female dogs (with three classic cases of extramural ureter ectopy and one atypical intramural ureter ectopy). The atypical intramural ureter was detected during a CT scan of a female dog. The ectopic ureter opened into a very short intramural section, which connected with the bladder wall at the border with the urethral orifice. Despite the short intramural section, the patient qualified for an extramural ureter procedure due to concerns over the risk of perforation and/or the lack of a clinical effect following cystoscopically guided laser ablation. Among all the dogs diagnosed with ureteral ectopia, nine were left-sided and seven were right-sided (Table 1).

The intramural ureter ectopy cases were qualified for cystoscopically guided laser ablation during diagnostic cystoscopy, which was performed right after CT. The mean operative time was 33 min, with a range of 28 to 37 min for cystoscopically guided laser ablation for female dogs. For male dogs, the mean operative time for cystoscopically guided laser ablation was 43 min, with a range of 41 to 46 min.

Laparoscopic ureteroneocystostomy was performed in cases of extramural ureter ectopy, for which there was no need to convert to open surgery. Intraoperative identification of the ureter was facilitated by observing its peristaltic movements whilst performing laparoscopic ureteroneocystostomy. From the first incision to the last suture, the mean operative time ranged from 41 to 60 min.

Since the owners had recorded whether urinary incontinence had started either postnatally or after purchase, it was possible to determine when the first episode of incontinence had occurred in each dog. No significant early or long-term complications were experienced by any of the dogs that underwent laparoscopic surgery. In one case, hematuria passing into the catheter was reported on the first day after the operation. A small amount of free fluid was present around the bladder in two cases, observed during follow-up examinations performed 24 h postoperatively; however, 48 h after the operation, the free fluid was no longer detectable. Two weeks after the procedure, intra-ureteral catheters were removed cystoscopically (Figure 4E,F). The skin sutures were removed on the tenth day after the procedure.

During the six-week ultrasound postoperative checkup, no long-term complications were detected. There were no recorded cases of effluence originating from the anastomosis or stenosis, leading to hydroureter or hydronephrosis, during the entire postoperative period. Healing by primary intention was observed in all postoperative wounds. Then, 24 to 48 h after the operation, all bladder catheters were removed, with the treated dogs passing urine on their own without signs of dysuria. Howbeit, urinary incontinence signs were still observed in one female dog after surgery; however, this animal responded to pharmacological treatment with phenylpropanolamine hydrochloride at a dose of 1 mg/kg bodyweight three times a day with food (Propalin, Vetoquinol, Poland).

The dogs treated via cystoscopically guided laser ablation had no long-term severe postoperative complications. Amongst the dogs in this group, three exhibited symptoms of cystitis within the first 10 days after surgery. The symptoms in all dogs disappeared after systemic antibiotic therapy with amoxicillin-containing clavulanic acid (Synergal Inj. 140 + 35 mg/mL ScanVet Poland) administered at a dose of 12.5 mg/kg for 10 days.

No abnormalities were present at the follow-up ultrasound examinations 6 weeks after surgery. At least six months after the operations, none of the dogs exhibited urinary symptoms, including urinary incontinence or infection, according to testimonies obtained from the owners.

## 4. Discussion

The results of this study indicate that regardless of the gender or type of ectopia burdening the affected dog, urinary incontinence due to ectopic ureters can be treated using minimally invasive surgery (MIS). This is the first report presenting the possibility of treating various types of ureteral ectopia in dogs using MIS techniques. Based on our experience in treating ureteral ectopia using MIS techniques and the presented cases, we suggest using cystoscopic laser ablation in cases of intramural ectopia with a ureteral course, as it allows for the ureteral orifice to be safely relocated to the urinary bladder. In contrast, extramural ectopic ureters and intramural ureters connecting to the bladder wall very close to the urethra should be treated laparoscopically.

According to the authors, most cases of open surgery can be avoided by utilizing MIS techniques; this particularly applies to veterinary clinics that perform laparoscopic and endourological procedures. Uroabdomen post-neocystoureterostomy has been noted as a major postoperative complication [17]. Throughout our study, irrespective of which surgical method was utilized, there were no noted major postoperative complications. Noël et al. [18] reported that there was a surgical revision rate of 11% (five dogs), including uroabdomen cases (three dogs) and cases of colposuspension causing severe dysuria (two dogs), and major complications were experienced when using an open-surgical method for EU correction. Noël et al. [18] stated that anastomosis leakage at the site, cystotomy incision dehiscence, and leakage through the stay-suture site at the apex of the bladder are paramount factors with respect to uroabdomen. Trace hematuria after the first 24 h was noted as a minor complication in one of the female dogs after an operation. Other minor complications, such as dysuria, pollakiuria, or stranguria, were not documented. Compared to open-surgical approaches, this indicates a lower incidence of post-EU surgical complications. At the same time, the number of complications, both for laparoscopic correction and cystoscopically guided laser ablation, is similar to figures previously described in the literature [13,17].

Dekerle et al. [17] demonstrated that 72% of complications are reported to be minor, including hematuria. Post-neoureterostomy (open surgery) is associated with a greater incidence of lower urinary tract symptoms than cystoscopically guided laser ablation [17]. Prządka et al. [13], in their previous studies, found hematuria in 30% (3/10) of cases of postoperative complications resulting from laparoscopic correction in the treatment of ureteral ectopia in female dogs. The percentages of minor complications, as reported in clinical studies [18], are 37% (15 dogs), including dysuria (7 dogs, accounting for 47% of minor complications), pollakiuria (33%), and hematuria (20%), when using an open-surgical approach. Complications were reported in 32.2% of cases in a retrospective evaluation of the treatment of intramural ectopic ureters in female dogs using cystoscopically guided laser ablation; these included pollakiuria (16.1%), pigmenturia (9.7%), excessive vulvar licking (3.2%), and urinary tract infections (3.2%) [19]. In our research, cystitis was found in 3 out of 12 dogs, which responded to pharmacological treatment.

Both laparoscopic ureteroneocystostomy and cystoscopically guided laser ablation were feasible in all cases and did not require conversion. A similar result regarding the feasibility of laparoscopic correction was obtained by other authors for 10 procedures for the transposition of an ectopic ureter without the need to convert to open surgery.

In cases of cystoscopically guided laser ablation, Jacobson et al. [12] reported that anatomically correct positioning of the terminal ureteral orifice via transection of the medial wall was achievable in seven out of eight cases. Conversely, due to the presence of extramural EUs or extramural portions of EUs and injuries to the bladder mucosa secondary to cystoscopy, Hooi et al. [19] determined that EUs could not be corrected in 9.7% of cystoscopically guided laser ablation cases. Prządka et al. [13] applied the laparoscopic technique only to female dogs with intramural ectopic ureters and suggested that laparoscopic ureteroneocystostomy could be applicable in cases of extramural EUs. The results presented in this study show that laparoscopic ureteroneocystostomy in female dogs is a safe and feasible treatment option. After laparoscopic correction of EUs, urinary continence returned to 15/16 dogs in the present study. Despite proper correction of the ureter (confirmed via cystoscopy during intra-ureteral catheter removal) and the absence of lower urinary tract infections, one dog still presented signs of urinary incontinence. The aforementioned dog reacted to pharmacological treatment. In total, 35% of dogs displayed signs of recurrent incontinence in a follow-up study by Noël et al. [18], with the majority of the dogs responding to auxiliary treatment. According to reports, the postoperative continence rate after EU correction ranges from 22% to 72% [5,10,11,20,21,22,23,24]. Similarly, urinary continence rates of between 31% and 47% have been reported for female dogs treated via cystoscopically guided laser ablation [6,7]. Furthermore, in one study, urinary continence returned to three out of seven dogs as a result of the cystoscopically guided transection of intramural ectopic ureters alone [12]. Congenital urethral sphincter mechanism incompetence (USMI), disturbed urethral closure due to residual intramural EUs, hormone imbalances, hypoplasia of the bladder, inadequate surgery, lower urinary tract infections (UTIs), neurogenic abnormalities, a poorly developed trigone, recanalization of the ligated ureter, and vestibulovaginal stenosis are all factors that potentially influence either persistent or recurrent urinary incontinence following EU correction [2,4,11,22,24].

We made our own minor modifications to the minimally invasive techniques used. These modifications relate to the techniques used for cystoscopically guided laser ablation in female dogs and laparoscopic ureteroneocystostomy. In cystoscopically guided laser ablation, the changes included a lack of intraoperative catheterization and the use of contrast for the ectopic ureter and the use of only a holmium laser for ablation. The lack of an intra-ureteral catheter was compensated for by a minimally increased flow of sterile saline during cystoscopy, which allowed the ureter to remain open. Additionally, a ureteronephroscope was used during cystoscopy, enabling, in most cases, the performance of ureteroscopy, thus facilitating the identification of the course of the ureter and the subsequent ablation of its wall. The lack of a catheter in the ureter during cystoscopically guided laser ablation eliminated the risk of accidental damage to the catheter caused by holmium laser rays and the leaving of debris within the patients’ tissues. Cystoscopically guided laser ablation was also performed without an intra-ureteral catheter by Berent et al. using a flexible endoscope for male cases [16]. In laparoscopic ureteroneocystostomy procedures, in contrast to the originally described procedure, we routinely used an intra-ureteral catheter (Double Pigtail Ureteral Stent), which minimized the risk of possible stenosis at the site of anastomosis of the ureter with the bladder wall. These catheters were removed during cystoscopy two weeks after the surgical procedure. As we have reported, this approach prevents one of the most serious postoperative complications: stenosis and secondary obstruction of the ureter at the transplant site. Whilst treating ureteral stenosis, surgeons often utilize ureteral stents [25]; when used, complications are rarely observed after surgical correction of the EUs due to their practicality. Problems may occur during EU surgery, not least because of a lack of appropriate stent size for smaller dogs [26]. Among the dogs that underwent surgery, there were no instances of stenoses or anastomotic leakages. These are amongst the direst complications after the re-implantation of the ureter and have been documented in both human and veterinary medicine [18,25,27].

The average time for the laparoscopic surgery itself was less than 60 min. In our previous report about the possibility of using ureteroneocystostomy for the treatment of ectopic ureters in dogs, we demonstrated that this procedure took an average of 80 min [13]. At that time, we did not have the equipment required to facilitate cystoscopically guided ablation, and ureteroneocystostomy was a new procedure introduced into veterinary medicine. We have performed more surgical procedures for the treatment of ectopic ureters since then, so the lower surgical time was due to the learning curve. Among the dogs treated with cystoscopically guided laser ablation, the average procedure time was 33 min for females and 43 min for males. The cystoscopically guided scissor transection of intramural ectopic ureters had a median duration of 105 min, with a range of 40–170 min [12], while 113 min was the median range for the cystoscopically guided laser ablation of the ectopic ureters, with a range of between 57 and 280 min [19]. The shorter procedure time in our study on dogs treated with cystoscopically guided laser ablation is due to our use of a holmium laser. Another factor contributing to this shortened procedure time is the lack of catheterization of the ectopic ureter before the ablation of its wall. We completed the ablation of the ureter wall when it reached the urinary bladder lumen, which was determined based on macroscopic assessments during cystoscopy. Based on our own clinical experience, there is no need to ablate the ectopic ureter wall up to the vesicoureteral junction. The mere displacement of the ureteral orifice into the bladder lumen is sufficient to achieve clinical improvement without subsequent symptoms of urinary incontinence due to ureteral ectopia. To achieve this, it is necessary to perform a detailed evaluation of CT scans with intravenous contrast prior to cystoscopy. Doing so allows one to identify the route of the ureter and decide which surgical method is appropriate, especially in cases of intramural ureters connecting to the bladder wall at the border with the urethra. In such cases, laparoscopic ureteroneocystostomy treatment is justified despite the short course of the intramural ureter, which was the case for one of the female dogs described in this manuscript. This method prevents perforation of the bladder wall at the junction of the ureter and the bladder or ineffective ablation of the ureter wall.

One of the factors limiting the generalization of the results of this study is its small sample size. Only dogs treated laparoscopically were subjected to repeated cystoscopic examination during the removal of the intra-ureteral catheter. The repetition of cystoscopy to allow for a proper assessment of the healed ureter and any signs of stenosis to the ureteral orifice would have been ideal. Conversely, as stated by Jacobson et al. [12], it would be difficult to provide justification for additional procedures given that most dogs were faring well and improvements could be seen. Clinical observations, based on information obtained from owners by telephone, were made for up to six months. Additionally, these results require confirmation through larger studies. Any divergence in long-term outcomes between the available treatment routes for ectopic ureters must be determined in prospective comparative studies. Studies determining the safety and effectiveness of laparoscopic ureteroneocystostomies for the treatment of male dogs presenting with extramural ectopic ureters are still necessary.

Nevertheless, this study reveals the possibility of treating ureteral ectopia in dogs using minimally invasive techniques, regardless of gender, with the choice of surgical technique depending on the type of ectopia presented. However, the possibility of applying the laparoscopic technique to treat male dogs with extramural ectopia of the ureter requires investigation. We believe that such a procedure will also be possible.

## 5. Conclusions

Laparoscopic ureteroneocystostomy and cystoscopically guided laser ablation are safe and effective minimally invasive surgical techniques, providing an alternative to open surgery for the treatment of urinary incontinence due to intramural ectopy of the ureters in female dogs. The only significant limitation of the presented minimally invasive techniques is the need to have appropriate equipment and skills in performing advanced laparoscopic and endourological procedures. At the same time, in clinics that meet the above requirements, it is our belief that all cases of ectopic ureters with clinical symptoms should be treated with one of the minimally invasive surgical techniques. Cystoscopically guided laser ablation should be considered in cases of intramural ectopy of the ureter, while laparoscopic ureteroneocystostomy is indicated for cases of extramural ectopy of the ureter or intramural ectopy when cystoscopically guided laser ablation cannot be used. Ultimately, it is worth remembering that in cases of possible complications, conversion to open surgery can always be performed, regardless of the minimally invasive technique used.

## Figures and Tables

**Table 1:** Additional information about the treated animals.

	Dog Breed	Age (Months)	Weight (kg)	Sex	Ectopic Type	Side	Type of Procedure	Surgical Procedure Time (Minutes)
1	Mix	8	9	Female	Intramural	Left	Cystoscopy-guided laser ablation	32
2	Mix	10	15	Female	Intramural	Left	Cystoscopy-guided laser ablation	37
3	Mix	15	12	Female	Intramural	Left	Cystoscopy-guided laser ablation	28
4	Entlebucher	12	25	Female	Intramural	Right	Cystoscopy-guided laser ablation	31
5	Golden retriever	11	26	Female	Intramural	Left	Cystoscopy-guided laser ablation	33
6	Golden retriever	14	23	Female	Intramural	Right	Cystoscopy-guided laser ablation	32
7	Mix	16	8	Female	Intramural	Left	Cystoscopy-guided laser ablation	35
8	Entlebucher	24	26	Female	Intramural	Right	Cystoscopy-guided laser ablation	36
9	Golden retriever	7	17	Female	Intramural	Left	Cystoscopy-guided laser ablation	33
10	Mix	9	16	Female	Intramural caudal (Classified extramural)	Right	Laparoscopic ureteroneocystostomy	60
11	Mix	9	14	Female	Extramural	Left	Laparoscopic ureteroneocystostomy	41
12	Mix	8	17	Female	Extramural	Right	Laparoscopic ureteroneocystostomy	49
13	Golden retriever	12	31	Female	Extramural	Left	Laparoscopic ureteroneocystostomy	58
14	Mix	10	23	Male	Intramural	Right	Cystoscopy-guided laser ablation	42
15	Golden retriever	14	28	Male	Intramural	Left	Cystoscopy-guided laser ablation	41
16	Entlebucher	8	20	Male	Intramural	Right	Cystoscopy-guided laser ablation	46

## Data Availability

Data are contained within the article.
